# Use of laser in inter-disciplinary dentistry: An *in-vivo* study

**DOI:** 10.6026/973206300210054

**Published:** 2025-01-31

**Authors:** Debkant Jena, Mukesh Soni, Swati Solanki, Mir Hasan Ansari, Rahul Anand Razdan, Alekhya Repudi

**Affiliations:** 1 Department of Conservative dentistry & Endodontics, Kalinga Institute of Dental Sciences, KIIT University, Bhubaneswar, Odisha, India; 2 Department of Prosthodontics, Crown and Bridge, Govt College of Dentistry, Indore, Madhya Pradesh, India; 3 Department of Prosthodontics, Crown and Bridge, Govt College of Dentistry, Indore, Madhya Pradesh, India; 4 Private Practitioner, 3A dental and implant clinic, Delhi, India; 5 Department of prosthodontics, Crown and Bridge, Index Institute of dental sciences, Indore, Madhya Pradesh, India; 6 General Dentist, Hyderabad, Telangana, India

**Keywords:** Laser dentistry, periodontal therapy, endodontics, prosthodontics, interdisciplinary dentistry

## Abstract

The impact of laser technology on inter-disciplinary dental treatments, focusing on periodontal, endodontic and prosthodontic procedures is of interest. A sample of 15 participants received laser-assisted therapies and its outcomes such as healing time, pain reduction and microbial control were assessed. Data shows that laser application enhances tissue healing and improves procedural precision suggesting significant benefits for inter-disciplinary dental care.

## Background:

Laser technology has become an essential tool in dentistry, appreciated for its minimally invasive nature, precision and the ability to reduce discomfort during and after treatment [1]. Lasers offer unique benefits across dental specialties, enhancing clinical outcomes and reducing treatment times [2]. In periodontics, lasers are used effectively for managing soft tissue conditions, such as gingival inflammation, by targeting infected tissue while preserving healthy areas [3]. Studies have shown that laser-assisted periodontal therapy promotes faster wound healing and minimizes postoperative pain, making it a preferred option for soft tissue management [4]. Endodontic treatments also benefit significantly from laser technology, particularly in root canal decontamination. Research indicates that laser irradiation can enhance bacterial elimination from infected canals, resulting in improved disinfection outcomes compared to traditional methods [5]. Furthermore, lasers facilitate the cleaning of complex root canal systems that are often difficult to reach with mechanical instruments alone [6]. In prosthodontics, laser use contributes to precise soft tissue contouring around implants, enhancing aesthetic outcomes and supporting optimal healing at the implant site [7]. This application allows for improved integration of the prosthetic component with surrounding tissues, thus improving patient satisfaction and treatment longevity [8]. Therefore, it is of interest to describe the role of laser technology in interdisciplinary dental treatments, particularly as it relates to enhancing treatment efficiency and patient outcomes.

## Methods and Materials:

## Study design:

This *in-vivo* study was conducted to evaluate the effects of laser technology across different dental disciplines, including periodontal, endodontic and prosthodontic treatments. The study involved a sample size of 15 participants who met the inclusion criteria.

## Sample selection:

Participants were selected based on the following criteria:

[1] Adults aged 18-65 with clinical indications for periodontal, endodontic, or prosthodontic treatment.

[2] No history of systemic conditions that could interfere with wound healing or treatment response.

[3] Exclusion criteria included pregnancy, recent antibiotic use, or known hypersensitivity to laser treatment.

## Procedure:

Each participant received laser-assisted treatment tailored to their dental needs:

[1] Periodontal Treatments: Soft tissue laser therapy for managing gingival inflammation and promoting wound healing.

[2] Endodontic Treatments: Laser disinfection was applied in root canal therapy to enhance decontamination and reduce bacterial load.

[3] Prosthodontic Treatments: Soft tissue laser contouring around implants to improve integration and aesthetic outcomes.

All treatments were performed by licensed practitioners with specialized laser training. Laser parameters (wavelength, power settings and exposure time) were standardized across all procedures to maintain consistency.

## Data collection:

Data were collected for each participant immediately after treatment and at follow-up intervals of 1 week and 1 month post-treatment. The primary outcome measures included:

[1] Tissue healing time: Time taken for visible wound healing, assessed by visual inspection.

[2] Pain scores: Measured on a 10-point Visual Analog Scale (VAS).

[3] Inflammation reduction: Measured by gingival index scores in periodontal cases.

[4] Bacterial load reduction: Evaluated using microbial sampling for endodontic cases.

## Statistical analysis:

Descriptive statistics were calculated for all outcome measures, with means and standard deviations reported for continuous variables. The following statistical tests were used to assess treatment efficacy:

[1] Paired t-tests were performed to compare pre- and post-treatment pain scores and bacterial load within the same group.

[2] Wilcoxon signed-rank test was used for non-normally distributed data to assess changes in inflammation scores.

[3] ANOVA was conducted to evaluate differences across the three treatment types (periodontal, endodontic and prosthodontic) regarding healing time and patient satisfaction.

Statistical significance was set at p < 0.05. Data were analyzed using SPSS (Statistical Package for the Social Sciences), Version 25.

## Ethical considerations:

Ethical approval was obtained from the institutional review board. Informed consent was received from each participant before inclusion in the study.

## Results:

The results of this study are organized based on the primary outcome measures: tissue healing time, pain scores, inflammation reduction and bacterial load reduction across periodontal, endodontic and prosthodontic treatments (Table 1).

[1] Tissue healing time: Participants in the prosthodontic group demonstrated the shortest healing time (mean = 8.5 days) compared to the periodontal (10.2 days) and endodontic (9.8 days) groups.

[2] Pain score reduction: All groups reported significant pain reduction, with the periodontal group achieving the highest decrease in VAS pain scores (mean reduction = 6.5).

[3] Inflammation reduction: In periodontal treatments, inflammation was reduced by an average of 70% post-treatment, as measured by gingival index scores.

[4] Bacterial load reduction: The endodontic group demonstrated an 85.5% reduction in bacterial load, indicating effective laser decontamination of root canals.

The heat map below visually represents the outcomes for healing time, pain score reduction, inflammation reduction and bacterial load reduction, highlighting areas with greater clinical improvement (darker colors indicate better outcomes). The heat map displays the clinical outcomes across the different laser treatments in dentistry. The darker colors represent areas with better treatment outcomes, such as shorter healing times, greater pain reduction and significant bacterial or inflammation reduction, helping to visualize which treatment type benefited most in each outcome category (Figure 1 - see PDF).

## Discussion:

The results of this study demonstrate the effectiveness of laser technology in enhancing clinical outcomes across periodontal, endodontic and prosthodontic treatments. Each specialty observed improvements in tissue healing, pain reduction and, where applicable, microbial control. This section explores these findings in light of existing literature. The use of lasers in periodontal therapy demonstrated considerable reduction in gingival inflammation and shorter healing times as it lowers pain and speeds recovery, offering improved results in deep disinfection along with customized medical treatments [9]. Studies suggest that lasers enable precise targeting of inflamed tissues, preserving healthy areas and promoting a favorable healing environment [10]. This study supports prior findings that laser-assisted periodontal therapy can improve patient comfort and recovery rates, which aligns with research by Aoki *et al.* demonstrating reduced postoperative pain and inflammation in laser-assisted periodontal treatments [11]. In endodontics, the application of laser technology for root canal disinfection was particularly effective, achieving an 85.5% reduction in bacterial load. This is consistent with research indicating that lasers can penetrate deeper into root canal walls, reaching areas where traditional mechanical debridement may be less effective [12]. Gutknecht *et al.* found that laser decontamination significantly decreases microbial presence, enhancing the long-term success of root canal therapy [5]. Additionally, the non-invasive nature of lasers potentially reduces the risk of post-treatment complications, making it a promising adjunct to conventional endodontic procedures [2]. For prosthodontic applications, laser technology facilitated soft tissue management around implants, allowing for precise tissue contouring and integration. Kreisler *et al.* highlighted that lasers improve soft tissue healing around implant sites, which supports this study's findings of reduced healing times and favorable aesthetic outcomes [8]. The advantages of using lasers in implantology, such as minimal bleeding and reduced postoperative discomfort, are well-documented and likely contribute to enhanced patient satisfaction [7]. Overall, this study underscores the value of laser technology in interdisciplinary dental treatments. Dental practices continually advance to optimize patient experience and procedural efficiency, addressing the common perception of dental visits as a source of anxiety. These advancements include the integration of innovative technologies, such as laser dentistry, which offer numerous benefits for both patients and practitioners [13]. The benefits observed across different specialties demonstrate that lasers can enhance procedural accuracy, reduce recovery times and improve patient comfort. This aligns with the broader trend in dentistry towards minimally invasive, technology-driven treatments that prioritize both clinical efficacy and patient experience [4]. Lasers are versatile tools in modern clinics, employed in a wide range of applications from diagnostic procedures to complex surgical interventions and the processing of dental materials. Their ability to deliver highly concentrated energy offers significant advantages over traditional techniques, particularly when working with hard materials [14]. Future research should investigate larger sample sizes and long-term outcomes to further validate these findings and expand the scope of laser applications in dentistry.

## Figures and Tables

**Table 1:** Summary of key outcomes

**Treatment Type**	**Mean Healing Time (days)**	**Pain Score Reduction (VAS)**	**Inflammation Reduction (%)**	**Bacterial Load Reduction (%)**
Periodontal	10.2 ± 1.8	6.5 ± 1.2	70.0 ± 8.5	-
Endodontic	9.8 ± 1.5	5.8 ± 1.4	-	85.5 ± 7.3
Prosthodontic	8.5 ± 2.0	6.2 ± 1.0	-	-
